# Gynecomastia and hypertension in a patient treated with posaconazole

**DOI:** 10.1002/ccr3.3376

**Published:** 2020-09-28

**Authors:** George R. Thompson, Prasanth N. Surampudi, Alex Odermatt

**Affiliations:** ^1^ Department of Internal Medicine Division of Infectious Diseases University of California Davis Medical Center Davis CA USA; ^2^ Department of Internal Medicine Division of Endocrinology University of California Davis Medical Center Davis CA USA; ^3^ Swiss Centre for Applied Human Toxicology and Division of Molecular and Systems Toxicology Department of Pharmaceutical Sciences University of Basel Basel Switzerland

**Keywords:** 11beta‐hydroxylase, adverse drug effect, estradiol, gynecomastia, hypertension, hypokalemia, posaconazole

## Abstract

Posaconazole therapy may lead to increased serum estradiol levels and development of gynecomastia. Early detection by endocrine hormone measurements may help preventing gynecomastia.

## Graphical abstract

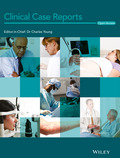

## 1 INTRODUCTION

We describe a patient with gynecomastia and pseudohyperaldosteronism caused by posaconazole treatment of pulmonary coccidioidomycosis infection. Gynecomastia has not previously been reported in posaconazole therapy. Substitution of posaconazole by voriconazole reversed blood pressure and serum potassium while gynecomastia remained unchanged. Early detection by endocrine measurements may help preventing gynecomastia.

Adverse drug effects account for up to 20% of all cases of gynecomastia.^1^ Drugs can disrupt hormonal regulation, thereby affecting ductal growth, alveolar differentiation, and subcutaneous fat deposition, which can contribute to male breast development. In particular, an increase in estradiol and progesterone levels with a concomitant decrease in testosterones can lead to gynecomastia. Drugs enhancing estrogen action promote ductal growth, medications increasing progesterone and prolactin levels can influence alveolar differentiation, and substances reducing androgen levels decrease their inhibitory effects on breast development and result in relatively increased effects of breast‐promoting hormones, thereby resulting in male breast development. Other hormones such as growth hormone (GH), insulin‐like growth factor (IGF‐1), prolactin, cortisol, and thyroid hormone act as permissive trophic factors and require an imbalance of estrogens and androgens for the development of gynecomastia. The sex steroids estradiol and progesterone require other mediators (eg, GH and IGF‐1) to sustain breast development.

The currently available azole antifungal agents used for systemic treatment are not fully selective, and besides blocking fungal ergosterol synthesis by inhibiting lanosterol 14α‐demethylase (CYP51), they can cause adverse effects by interfering with human steroidogenic cytochrome P450 (CYP) enzymes. This can lead to adverse drug‐drug and drug‐hormone interactions. The best‐studied example is the imidazole‐based antifungal compound ketoconazole, well known to cause gynecomastia, with an incidence of 4%‐8% at lower doses (200‐400 mg/day) and up to about 21% at higher doses (800‐1200 mg/day).^2^, ^3^, ^4^ These effects may be most frequent with long‐term therapy, subjecting patients to prolonged elevation of the estradiol/testosterone ratio. Ketoconazole has been found to markedly decrease serum testosterone concentrations, with a much smaller effect on estradiol, thereby significantly increasing the estradiol/testosterone ratio.^5^

Ketoconazole may alter estradiol and testosterone levels through multiple mechanisms. It was found to inhibit the adrenal 11β‐hydroxylase (CYP11B1), increasing the 11‐deoxycortisol/cortisol ratio by 15‐ to 80‐fold.^6^ Inhibition of this critical enzymatic step was found in some cases to be associated with gynecomastia, probably due to an enhanced adrenal androgen production because of inhibition of cortisol synthesis. Ketoconazole also inhibits 17α‐hydroxylase and 17,20‐lyase (both key steps in testosterone synthesis catalyzed by the same enzyme, ie, CYP17A1). As a result, less androgens are produced, and subsequently, also fewer estrogens are formed via aromatase (CYP19A1). Furthermore, ketoconazole can displace estradiol from sex hormone–binding globulin (SHBG),^7^ potentially resulting in increased estradiol levels. Additionally, ketoconazole can block androgen receptor binding of testosterone and dihydrotestosterone, thereby decreasing androgen signaling and altering the androgen‐estrogen balance toward the latter.^8^ These numerous “off‐target” effects relegated ketoconazole to the treatment of hypercortisolism and castration‐resistant prostate cancer while newer triazole (nonimidazole) antifungals have replaced its use in the treatment of systemic fungal infections.

The newer triazole antifungals (fluconazole, itraconazole, voriconazole, posaconazole, and isavuconazole) have not been reported to cause similar endocrinologic manifestations, despite nearly 30 years of clinical experience with these triazoles. A new formulation of posaconazole (delayed‐release tablets) has recently become available with resultant increases in serum drug concentrations compared to the previously prescribed oral solution formulation.^9^ This has been viewed as a favorable clinical development and has been a welcome addition to the antifungal armamentarium, given past studies finding improvements in posaconazole efficacy in the treatment of invasive fungal disease with higher serum drug concentrations.^10^ These higher levels, although potentially optimizing the efficacy, have recently been observed to be associated with adverse endocrinologic effects, specifically the pseudohyperaldosteronism caused by the inhibition of CYP11B1 and/or 11β‐hydroxysteroid dehydrogenase type 2 (11β‐HSD2).^11^, ^12^, ^13^ Although this adverse event has only recently been recognized and less than 20 cases have thus far been reported in detail,^14^ gynecomastia had previously not been observed in association with posaconazole‐induced pseudohyperaldosteronism.

## 2 CASE REPORT

A 38‐year‐old African American male with no prior past medical history was diagnosed with pulmonary coccidioidomycosis approximately 11 months prior to referral to the hospital. He initially had symptoms of fever, chills, chest pain, and weight loss, and was found to have a right lower lobe pneumonia and osteomyelitis of the right clavicle consistent with disseminated coccidioidomycosis. Laboratory results returned and were unremarkable with the exception of a positive *Coccidioides* complement fixation titer of 1:32 and a C‐reactive protein level of 254 mg/L. He was placed on oral fluconazole 600 mg/day. Over the next 8 weeks, he developed xerosis, cheilitis, and alopecia and refused further fluconazole therapy. He was subsequently transitioned to posaconazole delayed‐release tablets 300 mg/d. At return visit 5 months later, he was symptom‐free and the patient's coccidioidal complement fixation titer had decreased to 1:8. However, he had developed new onset of gynecomastia, supported by a mammogram showing benign fibrotic tissue, along with hypertension (blood pressure 155/98, heart rate 51). The mammogram did, however, not reveal any tumor. The normal human chorionic gonadotropin (hCG), follicle‐stimulating hormone (FSH), luteinizing hormone (LH), testosterone, and prolactin levels (Table 1) suggested the absence of prolactinoma or Leydig or Sertoli cell, and adrenal hormone–producing tumors. Furthermore, the patient had no nausea, diarrhea, or vomiting, and he was on no other medication and denied the use of any steroid supplements and estrogen‐rich foods.

**Table 1:** Laboratory values at the time of hypertension and gynecomastia evaluation

	Laboratory values	Reference range (see Ref. 16, 17)
Posaconazole level (µg/mL)	2.64	
Renin (ng/mL/h)	0.6	0.25‐5.82
Aldosterone (ng/dL)	**<1**	2.0‐18
11‐Deoxycortisol (ng/dL)	40	<158
Serum potassium	**3.3**	3.5‐5.0
Estradiol (pg/mL	**63**	<20
Cortisol (µg/dL)	11.1	5.0‐25
Cortisone (µg/dL)	1.8	1.0‐3.5
Cortisol/Cortisone	6.2	2‐8
TSH (μU/mL)	0.80	0.5‐4.7
T4 (free) (ng/dL)	1.1	0.8‐2.7
Prolactin ng/mL	8.8	0‐15
FSH (mIU/mL)	6.6	1.0‐12.0
LH (mIU/mL)	7.8	2.0‐12.0
Testosterone, total (ng/dL)	631	200‐1070
HCG (mIU/mL)	<2	<5

Values outside the normal range are shown in bold.

The development of posaconazole‐induced hypertension was further analyzed by laboratory testing (Table 1). As observed in previous cases,^11^, ^14^, ^15^ posaconazole‐induced hypertension was accompanied by hypokalemia and undetectably low aldosterone. In contrast to previous cases, renin was in the normal range, although at the lower end, and neither 11‐deoxycortisol level nor the cortisol‐to‐cortisone ratio was elevated. The other hormones, compared to reference ranges,^16^, ^17^ assessed were in the normal range, including testosterone; however, estradiol was significantly elevated.

Posaconazole therapy was then discontinued, and the patient was started on voriconazole 200 mg twice daily. The patient returned to clinic 4 months later, and at this time, his endocrinology laboratory values had normalized, with the exception of aldosterone that was now elevated at 31 ng/dL. However, his gynecomastia remained unchanged, although estradiol with 22 pg/mL was almost back to normal range. Since the discontinuation of posaconazole treatment, the patient is asymptomatic for almost 18 months now.

## 3 DISCUSSION

To our knowledge, this is the first case of posaconazole‐induced gynecomastia. However, we have noted elevated serum estradiol levels, but without signs of gynecomastia, in a previous case,^18^ suggesting that this adverse effect might be underestimated. Different mechanisms may explain the patient's elevated estradiol level. As a compensatory response to the inhibition of CYP11B1 by posaconazole, the adrenal steroidogenesis is enhanced and secreted adrenal androgens may be converted to estrogens in peripheral tissues. In this regard, CYP11B1 deficiency has been recognized as a cause of prepubertal gynecomastia, and enhanced adrenal androgen production with subsequent peripheral aromatization of androstenedione and testosterone is most likely responsible for the feminizing characteristics, although mineralocorticoids may also be involved.^19^, ^20^ Interestingly, biochemical evidence suggests that posaconazole can inhibit CYP17A1, mainly the lyase reaction,^12^, ^21^ which is needed for generation of both androgens and estrogens. Nevertheless, testosterone was normal in our patient while estradiol was significantly elevated (Table 1).

Alternatively, posaconazole, like ketoconazole, may result in decreased hepatic degradation of estrogens due to the inhibition of CYP3A4 and CYP3A7. A decreased conversion of estrone to 16α‐hydroxyestrone and estradiol to estriol may lead to elevated estrogen levels and a disturbed estrogen to androgen balance. Furthermore, follow‐on studies should also include enzymes involved in androgen and estrogen sulfation, and transporters of androgens and estrogens. Additionally, it remains unclear whether posaconazole decreases the binding of sex steroids to SHBG, leading to elevated free concentrations.

In contrast to previously described cases of posaconazole‐induced pseudohyperaldosteronism (PIPH) showing either elevated 11‐deoxycortisol, indicating CYP11B1 inhibition, or an increased cortisol to cortisone ratio, a biomarker of decreased 11β‐HSD2 activity,^11^, ^14^, ^15^, ^22^ this patient presented with 11‐deoxycortisol and a cortisol to cortisone ratio in the normal range (Table 1). As previously seen, aldosterone was undetectable but renin was at the lower end of the normal range. The serum posaconazole level was somewhat lower than in most previously reported cases of PIPH showing >3 μg/mL. Thus, the concentration achieved in our patient may not have been high enough to inhibit CYP11B1 and/or 11β‐HSD2 sufficiently to cause feedback stimulation, resulting in elevated serum 11‐deoxycortisol and/or cortisol to cortisone ratio. It needs to be noted that 24‐hour urine, which yields more sensitive information on changes in steroid homeostasis, was not available. The failure to detect aldosterone can be explained by the potent inhibition of aldosterone synthase (CYP11B2), which is more potently inhibited by posaconazole than CYP11B1.^12^, ^21^ The exact mechanism of hypertension and hypokalemia in this patient remains unclear. Enhanced sensitivity of the mineralocorticoid receptor by enhanced intracellular availability of corticosteroids or post‐translational modifications leading to enhanced receptor activation should be considered.

Nevertheless, substitution of posaconazole by voriconazole reversed the adverse effect on blood pressure, serum potassium, and endocrine hormones as seen in previous cases.^11^, ^14^ The fact that gynecomastia still persisted after discontinuation of posaconazole treatment is not surprising. Even with a return to normal estrogen levels, gynecomastia in males is frequently permanent as the breast tissue undergoes fibrosis, as was seen in our case. This emphasizes the need for detecting disturbances of endocrine hormones as early as possible during posaconazole treatment.

In conclusion, we report the first case of posaconazole‐induced gynecomastia and review the known adrenal enzymatic pathways responsible for the development of drug‐induced gynecomastia. Future work further delineating the exact pathophysiologic mechanism(s) behind posaconazole‐induced gynecomastia should be undertaken to more fully understand what might be an underreported phenomenon.

## CONFLICT OF INTEREST

None declared.

## AUTHOR CONTRIBUTIONS

GRT and PNS: assessed patient data. GRT, PNS, and AO: designed the concept of the manuscript, performed the literature search, defined the intellectual content, and wrote the manuscript.

## ETHICAL APPROVAL

This study was approved by the institutional review board of the University of California, Davis School of Medicine, Davis, USA. The patient gave consent to the study but was lost for follow‐up.
